# Pediatric Cardiology Echocardiography Curriculum and Workshop for Pediatric Residents in Mwanza, Tanzania-Using Deliberate Practice for Skill Acquisition

**DOI:** 10.1007/s00246-025-04140-9

**Published:** 2026-01-09

**Authors:** Shivani Govind Patel, Jennifer Acevedo, Angira Patel, Colleen Fant, Theophylly Mushi, Julieth Kabrigi, Tulla Masoza

**Affiliations:** 1 Division of Cardiology, Ann and Robert H. Lurie Children’s Hospital of Chicago, 225 East Chicago Ave. Box 21, Chicago, 60611 IL USA; 2 Division of Emergency Medicine, Ann, and Robert H. Lurie Children’s Hospital of Chicago, 225 East Chicago Ave. Box 21, Chicago, 60611 IL USA; 3 Department of Pediatric Cardiology, Jakaya Kikwete Cardiac Institute, Dar es Salaam, Tanzania; 4 Department of Pediatrics, Bugando Medical Center, Mwanza, Tanzania

**Keywords:** Pediatric echocardiography, Global health, Education, Africa, Resource-limited setting

## Abstract

Bugando Medical Center (BMC) in Mwanza, Tanzania, with two hundred pediatric beds, has a large burden of pediatric heart disease. The general pediatric residents are front-line cardiology providers. At the time of this study, there was a single pediatrician with echocardiography training, performing all echocardiograms and providing trainee education, teaching residents on-the-job with limited access to continued education. Our aim was to build pediatric residents’ cardiac ultrasound capacity at BMC through asynchronous learning and a hands-on workshop. The target learner group was BMC pediatric residents participating in a pediatric echocardiographic workshop. Baseline data was collected through a comfort survey, knowledge test, and skills test in acquisition/interpretation of echocardiographic images. An in-person, five-day workshop was conducted for acquisition of basic 2-dimensional images and recognizing abnormal images. At the end of the workshop, learners completed a knowledge post-test, an assessment of normal vs. abnormal images, and a skills test to determine ability to obtain standard images. 29 pediatric residents (15 PGY-2, 13 PGY-3, 1 registrar) participated in the workshop. The percentage of trainees who felt comfortable using an echocardiography machine increased from 60% to 96%. Knowledge test scores increased from 58% (*n* = 28) to 84% (*n* = 25) after intervention (*p* < 0.001). Skills assessment exhibited a significant improvement, from 10% to 83% of trainees’ ability to acquire images from all five imaging planes. Trainees were able to identify abnormal echocardiography images with an average score of 16 ± 3/20. An intense five-day echocardiography workshop significantly increased knowledge and skill in performing and interpreting basic pediatric echocardiograms for novice trainees and served as an excellent starting point for longitudinal future pediatric echocardiography training.

**Supplementary Information:**

The online version contains supplementary material available at https://doi.org/10.1007/s00246-025-04140-9.

## Introduction

Bugando Medical Center (BMC), located in Mwanza, Tanzania, is a tertiary referral hospital to Tanzania’s Lake and Western Zones and possesses a total catchment area of around 18 million people. BMC is an approximately 900 bed teaching hospital with about 200 beds dedicated to pediatrics, including a pediatric intensive care unit and a neonatal intensive care unit. Seventeen general pediatricians on faculty provide pediatric and subspecialty care, with assistance from a three-year pediatric residency program composed of 45 residents. A bidirectional partnership for pediatric residents between BMC and Lurie Children’s Hospital (LCH) has existed since 2006. As this partnership has grown, additional opportunities for resource sharing and collaboration have been unveiled, with one such priority area being advanced training in pediatric cardiology and echocardiography [1].

In 2023, there were no pediatric cardiologists at BMC despite a large burden of congenital and acquired heart disease in children [2]. General pediatricians provide pediatric cardiology clinical care, with front-line in-patient care, including performing echocardiograms, provided by pediatric residents. Medical care for acquired heart disease and early management of congenital heart disease is provided locally at BMC. This requires the residents and pediatricians providing cardiac care to be skilled in pediatric echocardiography. However, there is no formal training available, and residents mainly learn this skill on the go from pediatricians who have similarly learned from their seniors.

The primary goal of performing pediatric echocardiography at BMC is to have the knowledge and skills to obtain standard echocardiography images for a pediatric patient and be able to, (1) assess biventricular and valvar function, pericardial effusion, and evidence of pulmonary hypertension in patients without congenital heart disease; (2) diagnose simple lesions such as solitary patent ductus arteriosus, atrial septal defects, ventricular septal defects, Tetralogy of Fallot etc. to be able to provide appropriate medical care and consider timely tertiary care center referral; and 3) recognize complex or ductal dependent congenital cardiac defects such as D-transposition of the great arteries, single ventricle anatomy, coarctation of the aorta etc. that follow the palliative pathway.

Acquisition of echocardiographic imaging involves multiple skills, including understanding ultrasound physics, knowledge of how to use an ultrasound machine, memorizing protocols, and ability to build a 3-D mental model of cardiac anatomy based on 2-D tomographic images. A bootcamp approach has been used for training in transthoracic echocardiography (TTE) using multi-modality education (didactic lectures, demonstrations, and simulations) [3–7]. At LCH, the TTE bootcamp training is performed in a sequential, organized manner in which LCH pediatric cardiology fellows obtain exposure and understanding of echocardiography using a tomographic plane visualization tool (TOPLAV) [8], followed by knobology and instrumentation training on a cardiovascular echocardiography phantom, then protocol-based scanning on a cardiac simulator, and finally protocol-based scanning on a live model. Furthermore, all images acquired by trainees are stored on a virtual platform that allows educators to provide comprehensive feedback. The trainees are taught how to create standardized reports and provide time-sensitive communication to patients and clinical care providers. This program at LCH typically trains six learners per year with a minimum of four faculty and is carried out over a period of two weeks. Given that this same approach cannot be used in settings where there are limited technical and faculty resources as well as differing training objectives, program leadership from both LCH and BMC collaborated to develop a unique pediatric echocardiography workshop to meet the specific needs of pediatric residents at BMC in Mwanza, Tanzania.

**Aims**.

1. To build the cardiac ultrasound capacity of pediatric residents at Bugando Medical Center in Mwanza, Tanzania.
2. To host a cardiac ultrasound workshop at the BMC pediatric wards as part of a new pediatric cardiology training initiative.

## Materials and Methods

### Study Design and Setting

This was a pediatric echocardiography educational intervention implemented for pediatric residents at Bugando Medical Center (BMC) in Mwanza, Tanzania who voluntarily participated in our inaugural cardiac ultrasound workshop. All pediatric residents who had just started their 2nd or 3rd year of pediatric residency were included in this study, and intervention outcome was measured via knowledge surveys and skill assessments. The learning objectives of this cardiac ultrasound workshop were to, (1) Identify cardiac structures on a heart model and correlate echocardiogram images using the heart model, cardiac diagrams, and echocardiogram videos. (2) Review a specific protocol with a checklist of the basic echocardiographic images from various imaging planes and how to obtain them using visual anatomy cards and demonstration by faculty members. (3) Demonstrate knowledge in pediatric cardiac ultrasound and the ability to obtain echocardiographic images per defined protocol, (4) Interpret echocardiograms to recognize “normal” versus “abnormal” echocardiographic images.

## Intervention

This curriculum consisted of two parts: one, a curated list of resources for asynchronous learning provided online over a year (See Table 1), and two, an in-person, five-day workshop conducted at BMC.

**Table 1:** Asynchronous learning in the form of pre-recorded videos and other learning materials

	Topic	Purpose
1.	Cardiac anatomy	Review of basic cardiac anatomy and correlating echocardiogram pictures, using a heart model, diagrams, and echocardiography videos
2.	Pediatric echocardiogram protocol	Description of a complete pediatric echocardiogram protocol to help trainees understand the difference between a comprehensive pediatric echocardiogram and the focused pediatric echocardiogram that we aimed to train them
3.	Basic physics in ultrasound	To use the echocardiography machine most efficiently, including basic color and spectral Doppler
4.	Focused pediatric echocardiogram	Delineate a specific protocol with a checklist of the basic echocardiographic images from various imaging planes, accompanied by short videos demonstrating acquisition of these basic images
5.	Workbook for quick access and simple interpretation	A workbook introducing common echocardiogram reporting terminology and clinical interpretation for congenital heart disease, including assessment of ventricular and valvar function, diagnosis of coarctation of the aorta, D-transposition of the great arteries, and assessment of septal defects

The in-person workshop consisted of hands-on skill acquisition in scanning from the various imaging planes using heart models, echocardiography machines of several types, and human models (patients admitted to the hospital). The workshop was conducted over a five-day period, with three hours of hands-on time each day. The workshop schedule was designed to encourage interaction and build skills learnt the prior day, with the goal of performing a focused basic pediatric echocardiogram (See Table 2). Days 1 to 4 were devoted to training, and testing was performed on day 5. During the training hands-on portion, trainees worked in groups of 5–6, on parallel learning stations using either a handheld device, portable laptop-based device, or a full feature echo machine. Over days 1–4, it was ensured that each trainee could train on all three types of ultrasound systems. Each trainee worked directly with a faculty member to obtain correct images, with repetition as needed until successful completion of the worksheet tasks while the other five trainees watched.

**Table 2:** Workshop schedule for focused pediatric echocardiogram

	Day 1	Day 2	Day 3	Day 4	Day 5
**Didactic** Review imaging plane	Introduction Subcostal Imaging	Parasternal Imaging (Long and short axis)	Apical Imaging	Suprasternal Imaging	Testing on knowledge of all imaging planes
**Interactive** Review protocol with visual anatomy cards Demonstration followed by deliberate imaging sweep practice on mannequin, with verbalization of structures expected to be seen	Subcostal Imaging	Parasternal Imaging (Long and short axis)	Apical Imaging	Suprasternal Imaging	Testing on recognition of normal vs. abnormal echocardiogram images from all imaging planes
**Hands-on with live model** Demonstration followed by imaging sweep practice on live model, with identification of structures on echocardiography machine	Subcostal Imaging	Parasternal Imaging (Long and short axis)	Apical Imaging	Suprasternal Imaging	Testing on ability to demonstrate all imaging sweeps on live model, with identification of structures on echocardiography machines

## Measurement

Baseline data was collected from all pediatric residents who participated in the cardiac ultrasound workshop. This included a cardiac ultrasound education needs assessment form that collected their year of training, comfort level in performing a cardiac ultrasound, and personal perception of basic echocardiography knowledge and competency on a Likert scale (Online Resource 1, Comfort survey). Baseline knowledge in cardiac echocardiography was objectively determined with a pre-test which assessed knowledge in pediatric cardiac ultrasound using guidelines from the American Society of Echocardiography (Online Resource 2, Baseline knowledge test). A baseline skills assessment was also performed to assess the pre-bootcamp’s ability of residents to acquire basic 2-dimensional echocardiographic views from each imaging plane.

At the end of the educational intervention, the comfort survey and knowledge test were re-administered to learners. Additionally, a knowledge test on normal vs. abnormal echocardiogram recognition from each imaging plane was performed. This consisted of 30 echocardiographic clips, of which 10 were normal and 20 were abnormal, and trainees were asked to answer each echocardiographic clip if it was normal or abnormal. Finally, a skills assessment in acquisition of basic echocardiographic views from each of the imaging planes was completed. Skills assessment was performed on either a laptop-based or full-feature echocardiography machine. Skills assessment was performed for all trainees by only 2 faculty members to minimize inter-rater variability. These 2 faculty members have a combined experience of about 25 years as echocardiography educators for novice trainees at their home institution and had a shared mental model of the performance of standard pre-defined main (home-base) views, sweeps and pertinent structures that are seen on this imaging when adequately obtained. Three attempts were allowed to demonstrate the main view from each imaging plane, perform the sweep, and recognize pertinent structures seen. If a trainee was unable to demonstrate the required items, the educator, using a method of deliberate practice, provided immediate correction, followed by a repeat attempt by the trainee, for a total of three attempts for each imaging plane (Online Resource 3, Sample worksheet). For every item on the worksheet, the faculty rater evaluated if the trainee either performed the required task correctly, incorrectly, or was unable. The environment for this endeavor was a “fail-forward” approach. The short-term goal was to have each trainee demonstrate that they “know” (i.e., have knowledge) and “know how” (i.e., have knowledge of a skill), consistent with the bottom of Miller’s pyramid [9]. Training and assessment occurred in a psychologically safe environment, with trainees working in groups of approximately six people, each taking turns performing the worksheet tasks while the other five trainees watched. To maintain psychological safety, the faculty members performing the assessment did not report individual trainee performance to their residency leadership, and this also allowed for material repetition as residents called out and demonstrated the worksheet items.

### Statistical Analysis

We analyzed differences between baseline and post-test comfort and knowledge scores using descriptive Likert scale comparisons and paired t-tests, respectively. We also calculated scores on accurate recognition of normal vs. abnormal echocardiograms. Finally, we assessed trainee skill in acquiring basic echocardiogram images from each of the five imaging planes by documenting, for each imaging plane, the number of trainees who acquired the images at first attempt, number of attempts needed by trainees to obtain the required images, and number of trainees who were unable to obtain the required images after three attempts.

## Results

There were 29 participants, including 15 second-year pediatric residents, 13 third-year pediatric residents, and one registrar. The pre-participation comfort survey was completed by 28 trainees, and the post-participation comfort survey was completed by 24 trainees (See Table 3).

**Table 3:** Percentage of trainees at varying levels of comfort with echo imaging planes, cardiac anatomy, ultrasound physics, and knobology of an echocardiography machine before and after educational intervention

Comfort in understanding	Cardiac imaging & echo scanning planes		Cardiac anatomy		Ultrasound physics		Knobology and basic use of an echo machine	
Time of survey	Pre (*n* = 28)	Post (*n* = 24)	Pre (*n* = 28)	Post (*n* = 24)	Pre (*n* = 28)	Post (*n* = 24)	Pre (*n* = 28)	Post (*n* = 24)
Uncomfortable	21%	0%	11%	0%	25%	0%	32%	0%
Neutral	14%	4%	11%	0%	22%	17%	7%	4%
Comfortable	60%	95%	78%	90%	50%	79%	61%	96%

The survey also demonstrated that 20/28 trainees had performed and interpreted an echocardiogram before intervention. Knowledge test scores increased from 58% (*n* = 28) prior to intervention, to 84% (*n* = 25) after intervention (*p* < 0.001). Skills assessment of the 29 trainees in their ability to acquire images from five standard imaging planes exhibited a significant improvement, from only 10% of trainees able to acquire images from all five imaging planes before the workshop to 31% able at first attempt and 83% able at third and final attempt after the workshop. Table 4 displays the number of trainees who were able to demonstrate basic echocardiogram images from every imaging plane on each attempt.

**Table 4:** Number of trainees (percentage) able to successfully acquire images from each imaging plane

Imaging plane	First attempt	Second attempt	Third attempt	Within 3 attempts	Unable to obtain
Subcostal Coronal	22 (76%)	6 (21%)	1 (3%)	29 (100%)	0
Parasternal Long Axis	15 (52%)	6 (21%)	3 (10%)	24 (83%)	5 (17%)
Parasternal Short Axis	17 (59%)	9 (31%)	3 (10%)	29 (100%)	0
Apical	17 (59%)	9 (31%)	1 (3%)	27 (93%)	2 (7%)
Suprasternal Notch	23 (79%)	4 (14%)	2 (7%)	29 (100%)	0
All imaging planes	9 (31%)	10 (34%)	5 (17%)	24 (83%)	5 (17%)

Additionally, assessment of the 29 trainees in recognition of normal vs. abnormal echoes yielded an average score of 21/30 with a standard deviation of four. Of the 30 questions, normal echocardiography findings were accurately recognized with an average score of 5/10 and a standard deviation of two, while abnormal echocardiography findings were accurately recognized with an average score of 16/20 and a standard deviation of three.

## Discussion

### Pediatric Echocardiography Education

Our cardiac ultrasound curriculum consisted of an asynchronous learning provided online over a year and an in-person, five-day workshop conducted at BMC. The trainees that participated in this curriculum were able to improve their test scores from 58% prior to intervention, to 84% after intervention (*p* < 0.001). Skills assessment of the trainees in their ability to acquire images from five standard imaging planes exhibited a significant improvement, from only 10% of trainees able to acquire images from all five imaging planes before the workshop to 83% of trainees able to acquire them at third and final attempt after the workshop. Moreover, the trainees were able to recognize abnormal echocardiography findings accurately with an average score of 16 ± 3/20. Our study demonstrates that novice trainees can be effectively prepared to obtain 2-dimensional echocardiogram images on any echocardiography machine in a short duration of time using a rapid cycle deliberate practice, fail-forward approach. Performing a transthoracic echocardiogram is one of the most valuable skill sets acquired by front-line pediatric cardiology providers. There is a very steep learning curve in acquiring this skill set, which includes understanding ultrasound physics, learning how to use an ultrasound machine, memorizing a protocol, and building a 3-D mental model of cardiac anatomy based on 2-D tomographic images. Typically, this training is undergone over several weeks or months by cardiac sonographers and advanced training fellows who provide subspecialty care in pediatric cardiology. At a minimum, most academic pediatric cardiology centers use the concept of an “echocardiography bootcamp” to initiate the cardiac ultrasound training for novice trainees [10]. This is typically done over a period of two weeks for several hours each day using didactic sessions, simulation, and live model use, followed by several years of ongoing training, with the objective of diagnosing complex congenital heart disease with detailed anatomical and morphological details as to align with the surgical, therapeutic, or palliative pathway required for each patient. The basic and initial steps to perform and interpret echocardiograms are the same for front-line pediatric cardiology providers in all settings. Thus, pediatric residents in resource-limited settings who need to recognize, diagnose, and prognosticate congenital heart defects as well as assess cardiac function, effusion, and evidence of pulmonary hypertension need the same level of initial training allowing them to provide pediatric cardiology care to their patient population.

## Resource Limitations and Differences

Bugando Medical Center (BMC) is a busy zonal referral hospital with about 200 beds dedicated to pediatrics, with a large population of pediatric cardiac disease and pediatric residents and pediatricians serving as the primary providers for children with cardiac disease. Pediatric cardiology care at BMC is impacted by a limited availability of resources, including unavailable prostaglandins, no local availability of interventional cardiology or pediatric cardio-thoracic surgeons, and cost-prohibitive transfers to the Jakaya Kikwete Cardiac Institute (JKCI) that serves over 61 million people, and is located 1,138 km from lake zone, and western regions (an approximately two-hour flight or 18-hour bus ride). The highest level of cardiac care available in the country is currently limited to surgical repair of simple cardiac defects and excludes any complex biventricular repair or single ventricle surgical palliation [11].

The training in pediatric cardiology needed for BMC’s pediatric residents is unique and different than what is provided to residents in western settings that have more access to subspecialty expertise, do not have to perform echocardiograms due to sonographer support, and have management pathways that typically veer towards early surgical care. The ultrasound systems that may be available to use for educational and clinical use may include a variety of instrumentation including but not limited to handheld devices, portable laptop-based echo machines, or full feature echo machines. Our trainees at BMC required a unique echocardiography protocol that specifically met the needs of their institution and the patient population they served. This curriculum differed from a typical point of care ultrasound or a focused echocardiogram curriculum but was instead a standard echocardiogram from all basic imaging planes with acquisition and interpretation skills that matched the requirements of the typical management plans for their cardiology patients.

Our hands-on workshop included training on handheld devices that were brought by Lurie to BMC; a portable laptop based machine that was borrowed from the intensive care unit at BMC, which served as the device that residents used to scan patients on call; and a full feature echo machine that was used by the clinical team to perform echocardiograms in the outpatient center. We ensured that each trainee was able to learn basic instrumentation for all three types of devices used. The ability to include handheld devices made it feasible to implement multiple parallel training stations which allowed for increased educational time spent on individual trainees by the faculty member. In addition, the trainees were able to be trained and tested on the ultrasound systems that were locally available to them, ensuring that they became comfortable and competent in using those machine systems.

Image archiving, standardized reporting and communication with patients and clinical teams are foundational elements of the practice of echocardiography and are typically included in our echocardiography training curriculum at our institution within the US. However, the exact same resources are not available at BMC. There is a lack of a data storage system that would allow for long term image archiving, precluding retrospective review of image analysis, and more robust quality improvement initiatives. The reporting system includes a standard word document template that has been created by the pediatricians that provided pediatric cardiology care, to generate an electronic copy of the echocardiogram report that is printed and placed in the patient’s medical chart. Images acquired by the trainees are stored on the machine temporarily until the supervising attending reviews them. A verbal report with pertinent findings is made by the residents to the supervising attending. In case of a clinical emergency, video calling mobile applications are used by the trainee to share videos of the acquired images with the supervising physician. Once the attending physician reviews images, a preliminary report with brief findings is written by the trainee in the medical chart. The attending physician typically performs additional imaging as needed, followed by a final report per the standard template by the attending. With these resource limitations at BMC, we focused on image acquisition and interpretation, allowing the local teams to continue their usual process in image storage, reporting and communication to patients and clinical providers. With the arrival of a full-time pediatric cardiologist at BMC, there has been a significant improvement in the amount and quality of immediate feedback provided to the trainees on their echocardiography skills. This is in addition to the feedback provided to them in the monthly virtual sessions conducted by our team.

### Impact of Echocardiography Education in A Resource-Limited Area

It has been shown that, in resource-limited settings, focused echocardiography training may be a reasonable alternative to subspecialty cardiovascular echocardiography training [12]. Moreover, Acheampong et al. [13] demonstrated that limited echocardiographic studies performed by non-cardiologists can increase access, improve diagnosis, and allow for earlier medical therapy. A recent scooping review on ultrasound training in low resource settings provided best practice recommendations that included focused hands-on training, low trainer to trainee ratio, protected training time, online training options, use of local trainers, short and concise training manuals in print, continuous supervision and early and on-going evaluation, as well as tele-monitoring [14]. We were able to meet several of these best practice recommendations, especially the focused hands-on training, protected training time, online training, short and concise training manuals, as well as on-going supervision.

Pediatric residents who are front-line cardiology providers at large tertiary care centers which have limited resources must balance high volume and complex patient pathology in the context of limited resources such as lack of ultrasound machines, training, and subspecialty expertise. Being able to offer a pediatric cardiology curriculum with a focus on hands-on, in-person workshops to teach these novice trainees builds the cardiac ultrasound capacity of such programs, not only at the institutional level, but also in spreading the care to regional centers where these residents eventually practice pediatrics. In Tanzania, with a high prevalence of rheumatic heart disease [11], this skillset of performing basic echocardiogram allows pediatricians to diagnose highly prevalent rheumatic heart disease, with associated valvar dysfunction, early findings of heart failure from simple congenital heart defects, can lead to expedited referral and eventually improved care. It also allows them to recognize complex defects and triage them into the palliative route, given the non-availability of surgical intervention for complex defects. In the process of creating and disseminating this curriculum, other collaborative learning and clinical opportunities are uncovered, including but not limited to collaboration with local adult cardiology training programs/resources, regional tertiary care centers that can provide more frequent hands-on training, and international ongoing education programs via asynchronous and virtual training sessions. The goal of creating and conducting a workshop to teach basic echocardiography is to not only educate novice trainees, but also to establish a smaller group of super-users, and, using the concept of train-the-trainer, create a self-sustaining educational model for future trainees. The workshop, as detailed in our manuscript, was the first step in initiating pediatric echocardiography training at BMC. Once we completed this initial workshop, there are several ways in which we have continued to provide structured follow-up and ongoing educational support for the team at BMC. To maintain knowledge, we continued our monthly virtual educational sessions where the residents from BMC would present a patient that one of them scanned, to which our sonographer educator would provide feedback on quality of imaging, and our cardiologist would aid in the clinical discussion of the case. With the newly learned skills of basic echocardiography, the residents overall were more comfortable and started scanning more patients on overnight calls, patients who would have typically waited until the next day. A self-selected subgroup of residents who were interested in pediatric cardiology, motivated to perform cardiac ultrasounds, improve their skills, and confident in their ability to scan patients became a valuable source of support for clinical care in cardiology. This subgroup also maintained an informal virtual channel with our sonographer educator to continue their learning. Unrelated to this workshop, but with the similar bigger objective of improving pediatric cardiology care at BMC, a newly trained pediatric cardiologist from Tanzania joined the faculty at BMC a few months after our workshop. This created a wonderful opportunity for the residents to have a local leader who would provide immediate feedback on their scans and further enhance the skillset of the subgroup of super-users. In less than a year after our first workshop, this pediatric cardiologist and 2 other residents from the super user group were able to spend a month at Lurie Children’s Hospital in the cardiology section as part of the long-term partnership that Lurie has with BMC. One of the graduated BMC residents who participated in this partnership has now also gone on to a separate cardiology fellowship. With their own pediatric cardiologist and continued support from Lurie Children’s Hospital we have continued to conduct training workshops annually and are in the process of creating a train-the-trainer program to build a sustainable pipeline to build cardiac ultrasound capacity in Mwanza, Tanzania.

### Limitations

There were several limitations to this study. This was a single site and there were a small number of learners available, < 30, and it is possible that these results would not be replicated in a different environment. Additionally, the learner pool was diverse, with some trainees having independently performed more prior echocardiography practice than others. Also, in this first iteration of the echocardiography bootcamp, most teachers were from LCH, not BMC, which may affect program acceptability and sustainability. As different institutions may have different resources available, the program would need to adjust to local resources. Moving forward, our future bootcamps/education plans to build the capacity of BMC pediatricians as echocardiography educators; thus, the LCH study team has engaged with a recently hired Pediatric Cardiologist, the first ever subspecialty trained pediatrician at BMC.

## Conclusions

An intensive echocardiography workshop can be performed to jump-start pediatric echocardiography training for novice trainees in a resource-limited setting.

## Supplementary Information

Below is the link to the electronic supplementary material.

Supplementary Material 1—Online resource 1 - Comfort Survey that assessed the comfort level of the trainees in performing a cardiac ultrasound, and their personal perception of basic echocardiography knowledge and competency on a Likert scale

Supplementary Material 2—Online resource 2 - Baseline Knowledge Test was a pre-test which assessed knowledge in pediatric cardiac ultrasound using guidelines from the American Society of Echocardiography

Supplementary Material 3—Online resource 3 - Sample worksheet for Skill Assessment and Recognition of Abnormal Echo

## Data Availability

The datasets generated during and/or analyzed during the current study are available from the corresponding author on reasonable request.
