# Supplementary material for: Pediatric Cardiology Echocardiography Curriculum and Workshop for Pediatric Residents in Mwanza, Tanzania-Using Deliberate Practice for Skill Acquisition
Source: Pediatr Cardiol. 2026 Jan 9;47(8):3432–9. doi: 10.1007/s00246-025-04140-9 (PMC13687231; doi:10.1007/s00246-025-04140-9)
Supplement: Supplementary file 1 [file 246_2025_4140_MOESM1_ESM.pdf]

Title: Pediatric Cardiology Echocardiography Curriculum and Workshop for Pediatric Residents in Mwanza, Tanzania – using Deliberate Practice for Skill Acquisition
Authors: Shivani Patel 1, Jennifer Acevedo 1, Angira Patel 1 , Colleen Fant 2, Theophylly Mushi 3, Julieth Kabrigi, 4 Tulla Masoza 4
1- Division of Cardiology, Ann and Robert H. Lurie Children's Hospital of Chicago, 225 East Chicago Ave. Box 21 Chicago, IL. 60611 U.S.A
2 - Division of Emergency Medicine, Ann and Robert H. Lurie Children's Hospital of Chicago, 225 East Chicago Ave. Box 21 Chicago, IL. 60611 U.S.A
3- Department of Pediatric Cardiology, Jakaya Kikwete Cardiac Institute, Tanzania
4 - Department of Pediatrics, Bugando Medical Center, Mwanza, Tanzania

Corresponding Author: Shivani Govind Patel, MBBS, MS, FASE, FACC. Email: shgpatel@luriechildrens.org

Transthoracic Echocardiography Workshop at Bugando - Comfort survey 2024

Assessment of comfort level of trainees with regards to echocardiography

1. Please state your name and role

   Enter your answer

2. How comfortable are you in your understanding of cardiac imaging and echo scanning planes

   - ○ Very comfortable
   - ○ Somewhat comfortable
   - ○ Neither comfortable nor uncomfortable
   - ○ Somewhat uncomfortable
   - ○ Very uncomfortable

3. How comfortable are you in your understanding of cardiac anatomy

   - ○ Very comfortable
   - ○ Somewhat comfortable
   - ○ Neither comfortable nor uncomfortable

- ◯ Somewhat uncomfortable
- ◯ Very uncomfortable

4. How comfortable are you in your understanding of ultrasound physics

- ◯ Very comfortable
- ◯ Somewhat comfortable
- ◯ Neither comfortable nor uncomfortable
- ◯ Somewhat uncomfortable
- ◯ Very uncomfortable

5. How comfortable are you in your understanding of an echo machine, its knobology and basic use

- ◯ Very comfortable
- ◯ Somewhat comfortable
- ◯ Neither comfortable nor uncomfortable
- ◯ Somewhat uncomfortable
- ◯ Very uncomfortable

6. Have you ever performed an echocardiogram

- ◯ Yes

◯ No

7. Have you ever interpreted an echo image

◯ Yes

◯ No

Never give out your password. Report abuse

Microsoft 365

This content is created by the owner of the form. The data you submit will be sent to the form owner. Microsoft is not responsible for the privacy or security practices of its customers, including those of this form owner. Never give out your password.

Microsoft Forms | AI-Powered surveys, quizzes and polls Create my own form

The owner of this form has not provided a privacy statement as to how they will use your response data. Do not provide personal or sensitive information. | Terms of use
