# Supplementary material for: Pediatric Cardiology Echocardiography Curriculum and Workshop for Pediatric Residents in Mwanza, Tanzania-Using Deliberate Practice for Skill Acquisition
Source: Pediatr Cardiol. 2026 Jan 9;47(8):3432–9. doi: 10.1007/s00246-025-04140-9 (PMC13687231; doi:10.1007/s00246-025-04140-9)
Supplement: Supplementary file 2 [file 246_2025_4140_MOESM2_ESM.pdf]

Title: Pediatric Cardiology Echocardiography Curriculum and Workshop for Pediatric Residents in Mwanza, Tanzania – using Deliberate Practice for Skill Acquisition
Authors: Shivani Patel 1, Jennifer Acevedo 1, Angira Patel 1 , Colleen Fant 2, Theophylly Mushi 3, Julieth Kabrigi, 4 Tulla Masoza 4
1- Division of Cardiology, Ann and Robert H. Lurie Children's Hospital of Chicago, 225 East Chicago Ave. Box 21 Chicago, IL. 60611 U.S.A
2 - Division of Emergency Medicine, Ann and Robert H. Lurie Children's Hospital of Chicago, 225 East Chicago Ave. Box 21 Chicago, IL. 60611 U.S.A
3- Department of Pediatric Cardiology, Jakaya Kikwete Cardiac Institute, Tanzania
4 - Department of Pediatrics, Bugando Medical Center, Mwanza, Tanzania

Corresponding Author: Shivani Govind Patel, MBBS, MS, FASE, FACC. Email: shgpatel@luriechildrens.org

Tanzania Transthoracic Echocardiography Workshop PreTest 2024

1

Please enter your name and role

Enter your answer

2

Which Imaging Plane is depicted in this picture (1 Point)

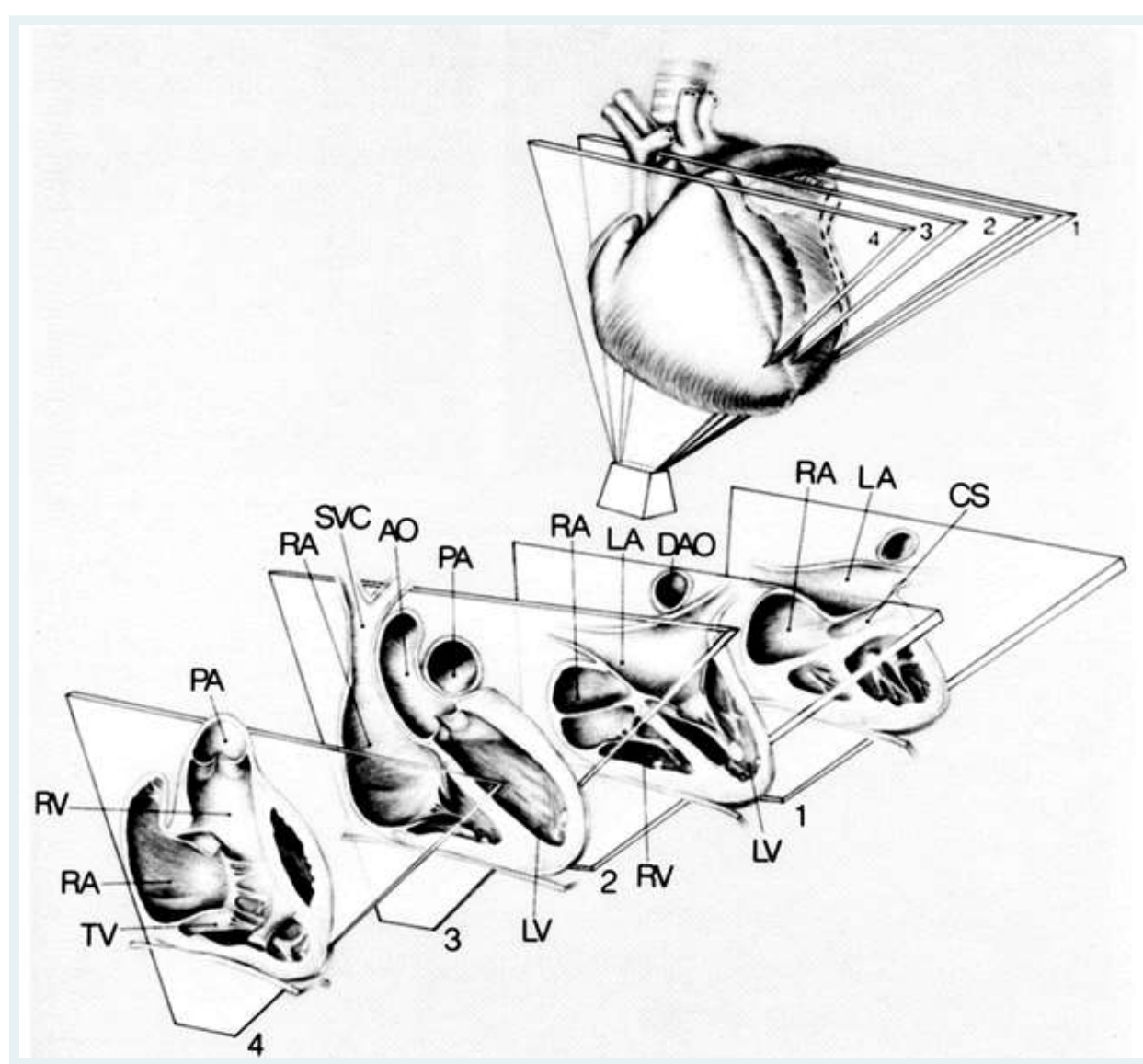

○ Subcostal Coronal

○ Subcostal Sagittal

○ Apical

Paraternal long axis

3

Which Imaging Plane is depicted in this picture (1 Point)

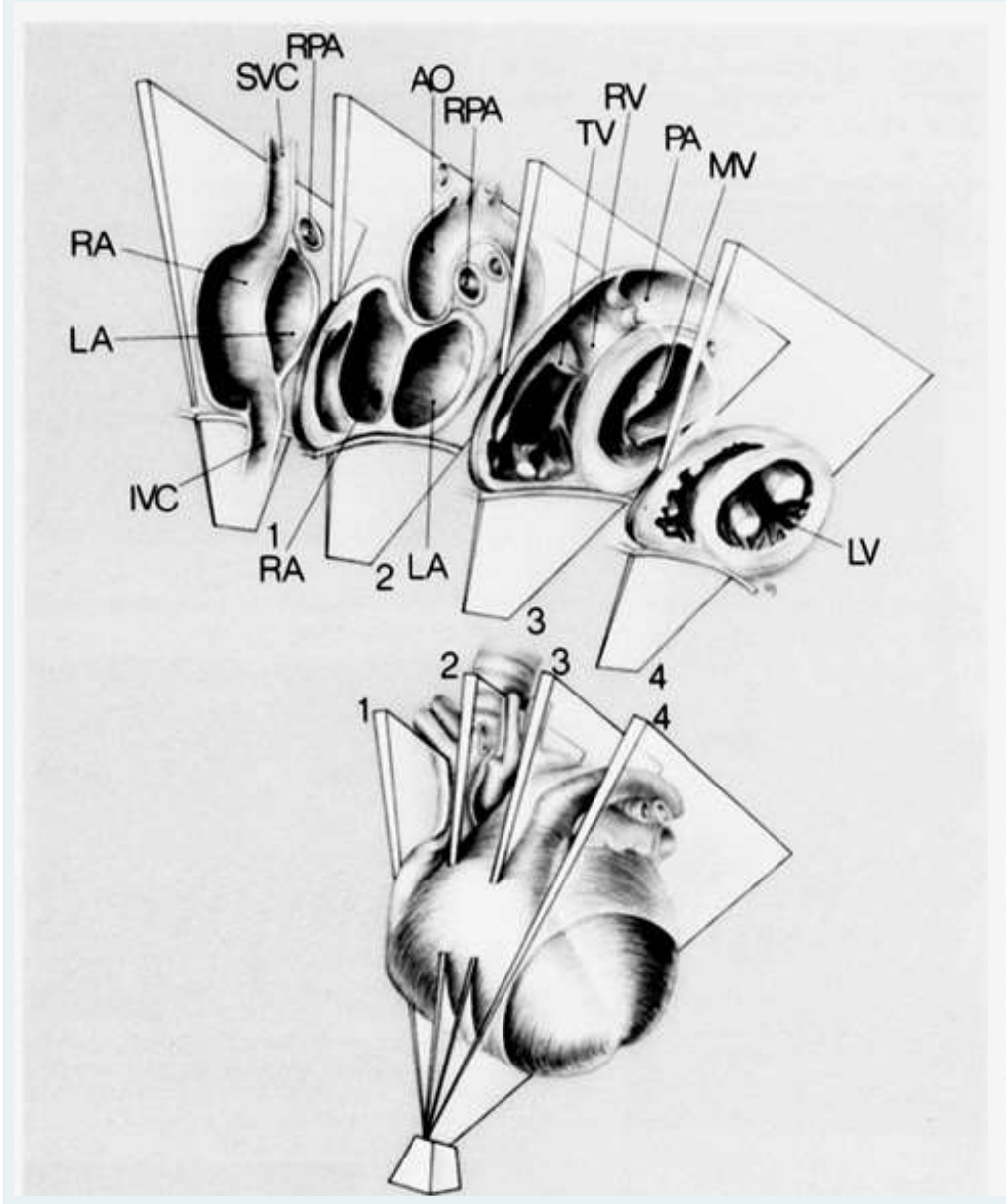

Subcostal Coronal

Subcostal Sagittal

Apical

Paraternal long axis

4

Which Imaging Plane is depicted in this picture (1 Point)

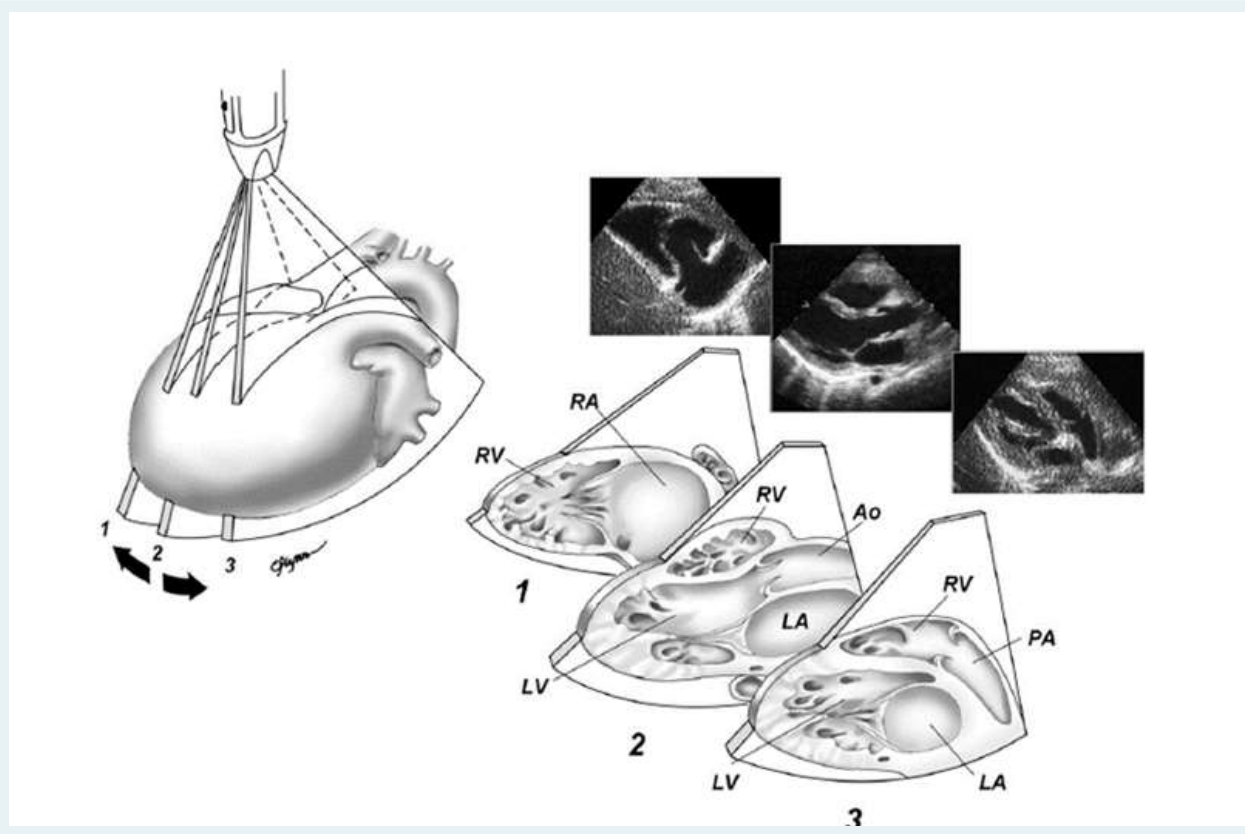

- Subcostal Coronal
- Subcostal Sagittal
- Apical
- Paraternal long axis

5

Which Imaging Plane is depicted in this picture (1 Point)

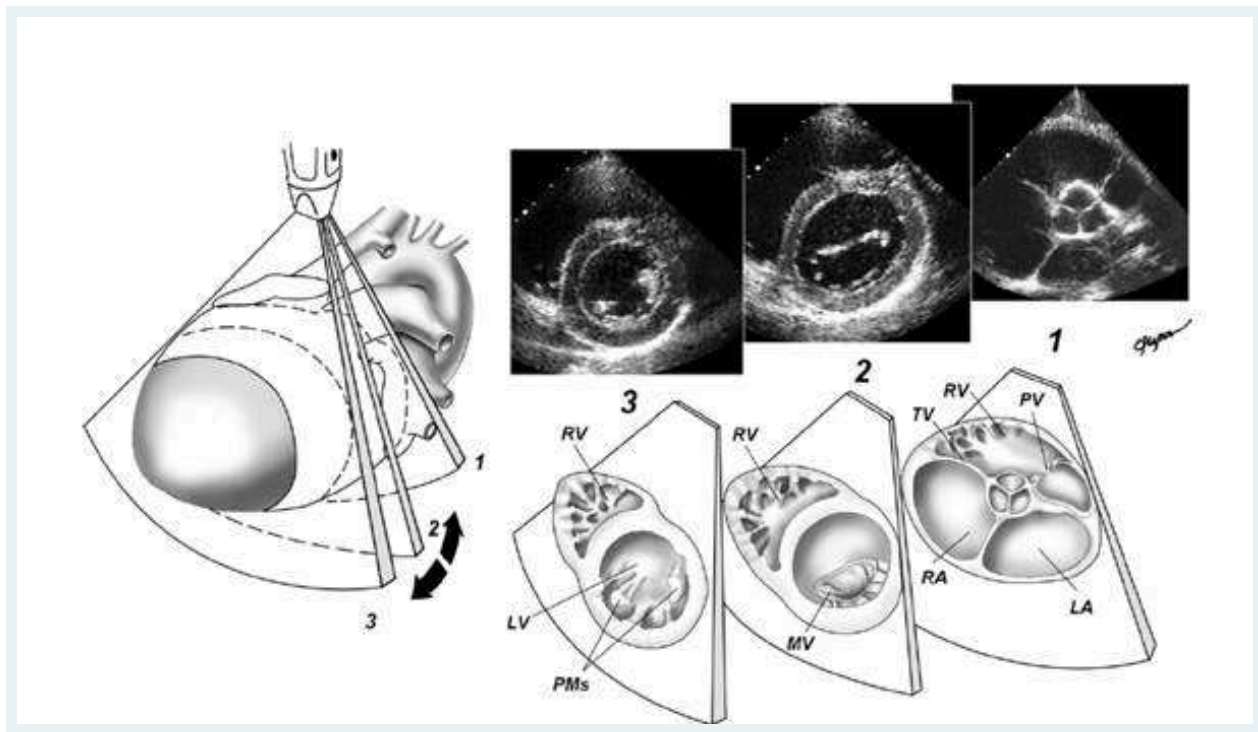

- Subcostal Coronal
- parasternal short axis
- Apical
- Paraternal long axis

6

Which Imaging Plane is depicted in this picture (1 Point)

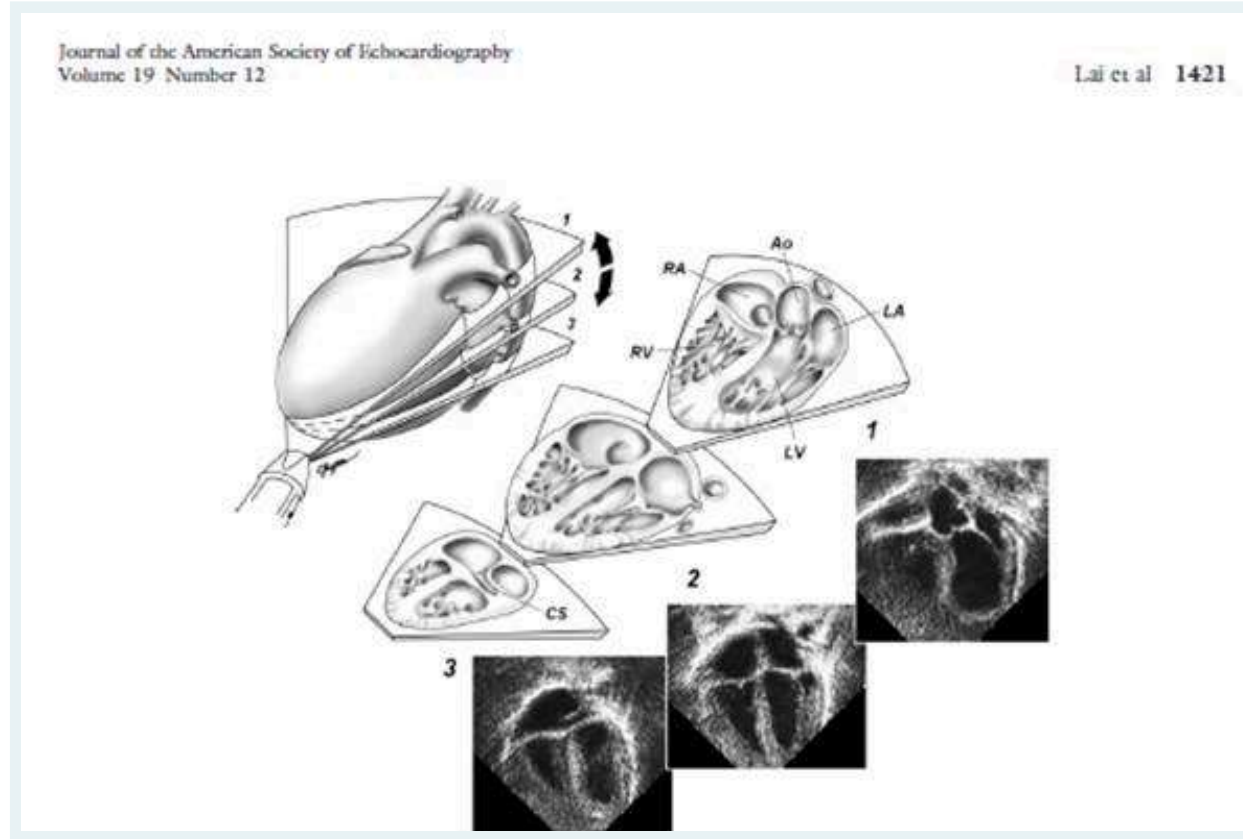

- ◯ Subcostal Coronal
- ◯ parasternal short axis
- ◯ Apical
- ◯ Paraternal long axis

7

Which Imaging Plane is depicted in this picture (1 Point)

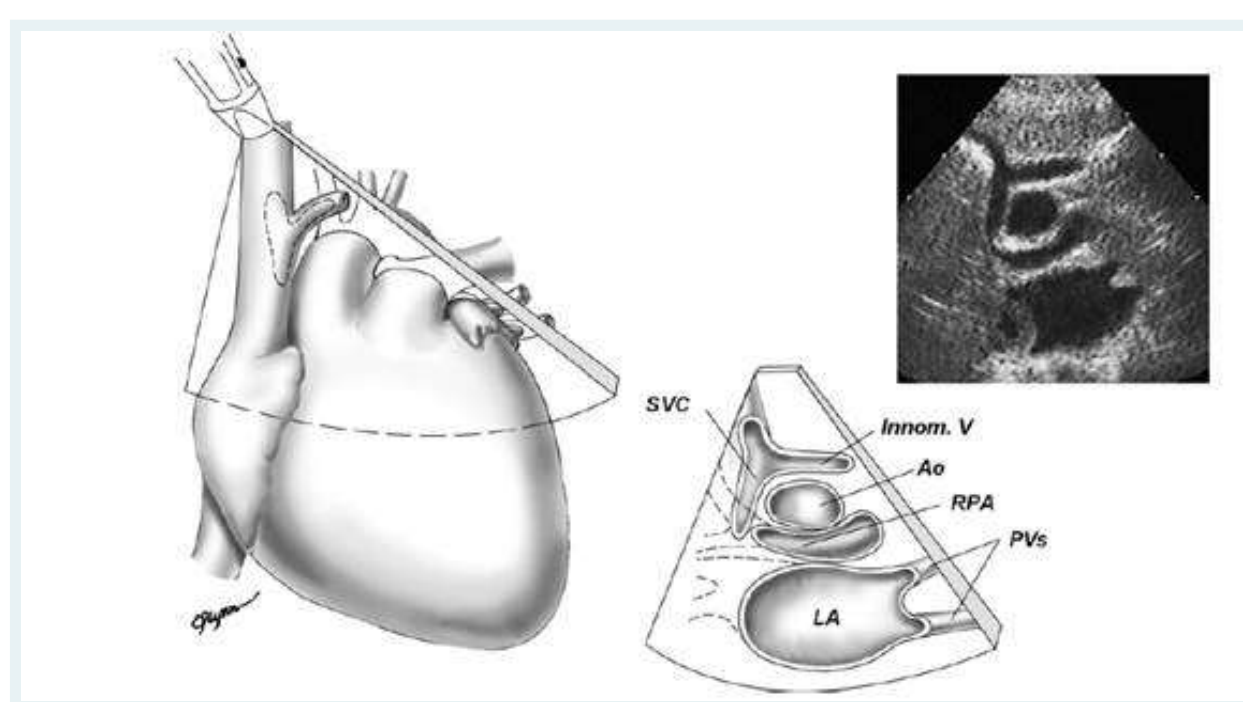

- ◯ suprasternal short axis
- ◯ parasternal short axis
- ◯ Apical

Paraternal long axis

8

This image from a subcostal view on a newborn represents (1 Point)

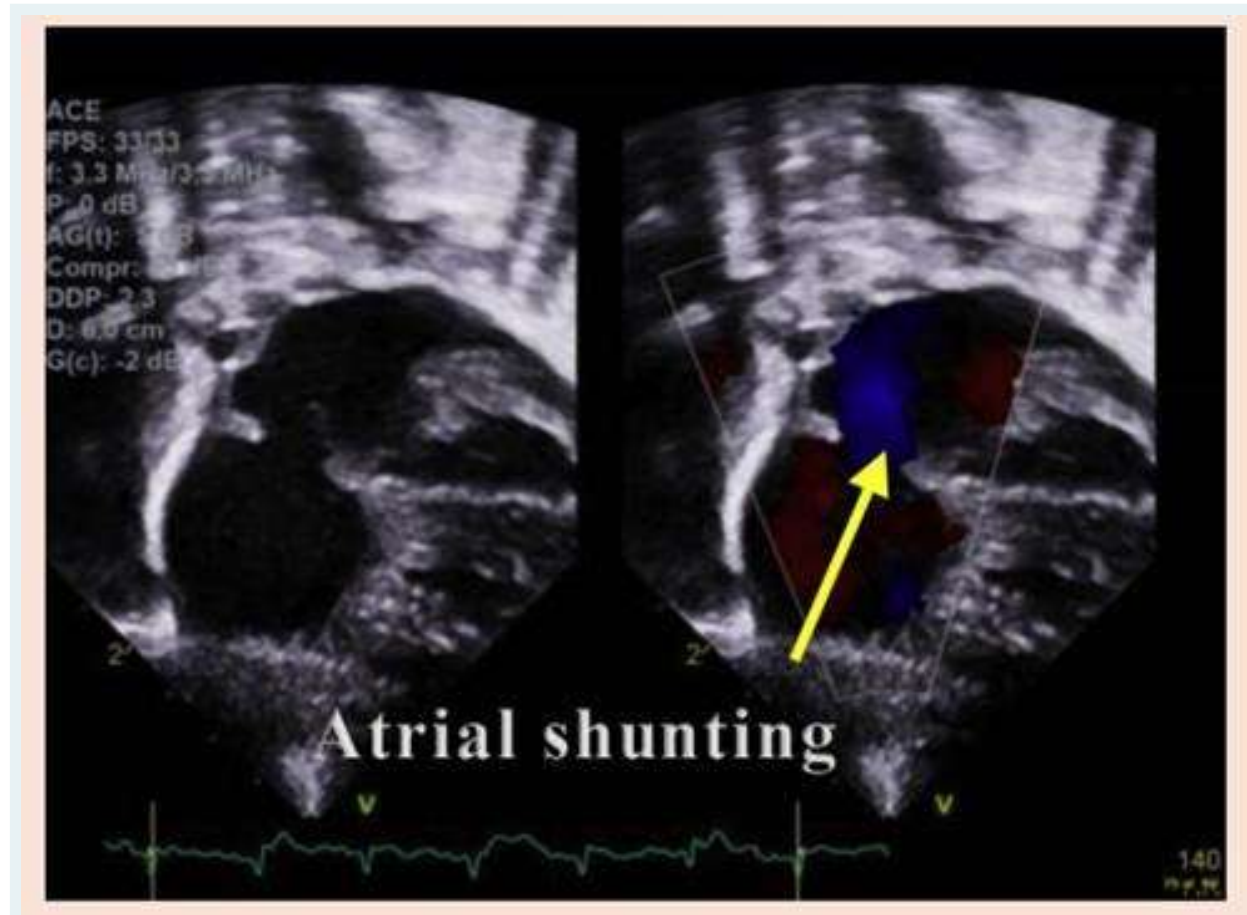

- a normal finding
- an abnormal finding

9

According to the American Society of Echocardiography, in a young child, the left ventricle should be measured at the region of the: (1 Point)

- A. Papillary muscles from a short axis image
- B. Aortic valve from a long axis image
- C. Mitral valve where only the anterior leaflet is seen from the long axis image
- D. Mitral valve where both the anterior and posterior leaflets are visualized from a short axis image

10

A patient has a blood pressure of 130/80 and a VSD jet of 5 m/sec. Assuming there is no aortic valve gradient, what is the right ventricular systolic pressure? (1 Point)

- A. 30 mmHg
- B. 80 mmHg
- C. 105 mmHg
- D. 125 mmHg
- E. 230 mmHg

11

A typical left aortic arch in the “candy cane view” is best seen with the transducer rotated with indicator pointed towards: (1 Point)

- A. 12 o’clock
- B. 1-2 o’clock
- C. 4-5 o’clock
- D. 7-8 o’clock
- E. 10-11 o'clock

12

Using a peak velocity of 4 m/sec across the pulmonary valve, estimate the severity of the pulmonary stenosis and its peak instantaneous pressure gradient.

(1 Point)

- A. Mild, 8 mmHg
- B. Mild, 16 mmHg
- C. Severe, 32 mmHg
- D. Severe, 64mmHg

13

What is a common 2D finding in a patient with severe PHTN? (1 Point)

- A. Round ventricular septal contour throughout the cardiac cycle
- B. Mild septal flattening in diastole
- C. Mild septal flattening in systole
- D. "D" sign due to flattening/bowing of interventricular septum in systole

14

Where should the probe indicator be when obtaining a standard PLAX view for a normally positioned heart? (1 Point)

- A. Towards the patient’s left shoulder
- B. Towards the patient’s right shoulder
- C. Towards the patient’s feet
- D. Towards the patient’s head

◯ E. Towards the liver

15

22. On Spectral Doppler, where is a positive Doppler shift depicted? (1 Point)

◯ A. Above the baseline

◯ B. Below the baseline

◯ C. Both above and below the baseline

16

This echo image demonstrates what subcostal imaging finding (1 Point)

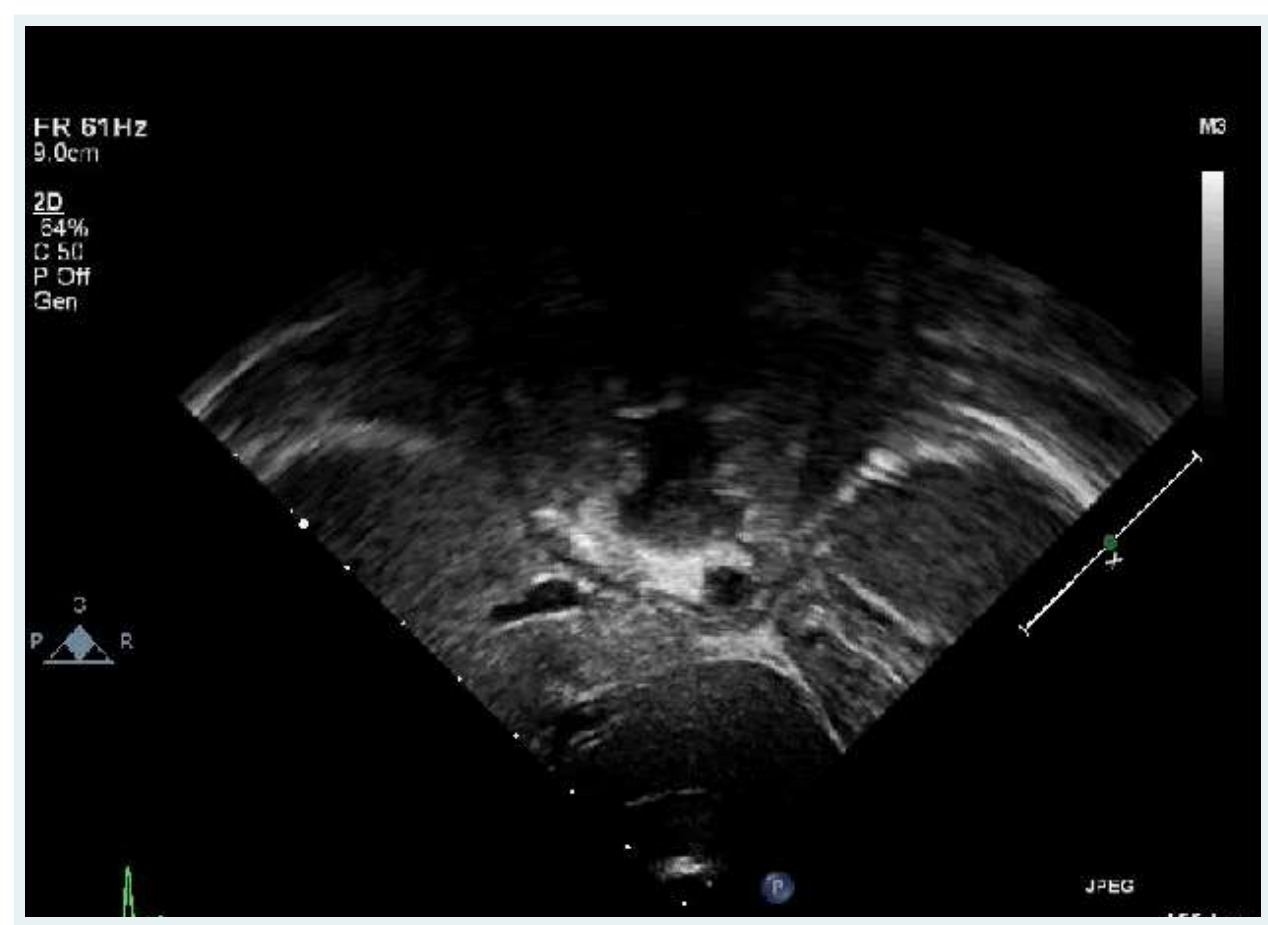

◯ A. Normal abdominal situs

◯ B. Abdominal situs inversus

◯ C. Interrupted IVC

◯ D. Left sided IVC

17

27. The normal orientation of the semilunar valves can be described as (1 Point)

- A. Aorta anterior and rightward of the pulmonary valve
- B. Aortic anterior and leftward of the pulmonary valve
- C. Pulmonary valve anterior and rightward of the aortic valve
- D. Pulmonary valve anterior and leftward of the aortic valve

18

29. Blood always flows from an area of _____ pressure to an area of _______pressure (1 Point)

- A. high--high
- B. low--low
- C. low--high
- D. high--low

19

30. What are energy or pressure differences called? (1 Point)

- A. Velocities
- B. Transducers

- C. Medians
- D. Gradients

20

Making the color box bigger increases the frame rate and resolution of your image. (1 Point)

- True
- False

21

From a parasternal imaging plane, the expected direction of flow in an isolated patent ductus arteriosus is (1 Point)

- A. away from the probe, consistent with L to R flow
- B. away from the probe, consistent with R to L flow
- C. towards the probe, consistent with L to R flow
- D. towards from the probe, consistent with R to L flow
- E. bidirectional flow

22

When assessing velocity of flow in a vessel by spectral Doppler, the ultrasound beam should be aligned (1 Point)

- A. Perpendicular to flow at 90 degrees
- B. Parallel to flow at 90 degrees
- C. Parallel to flow as close to 0 and no more than 20 degrees
- D. Parallel to flow at an angle >20 degrees
- E. None of the above

23

This view is helpful for looking at which of the following structures, especially in older children? (1 Point)

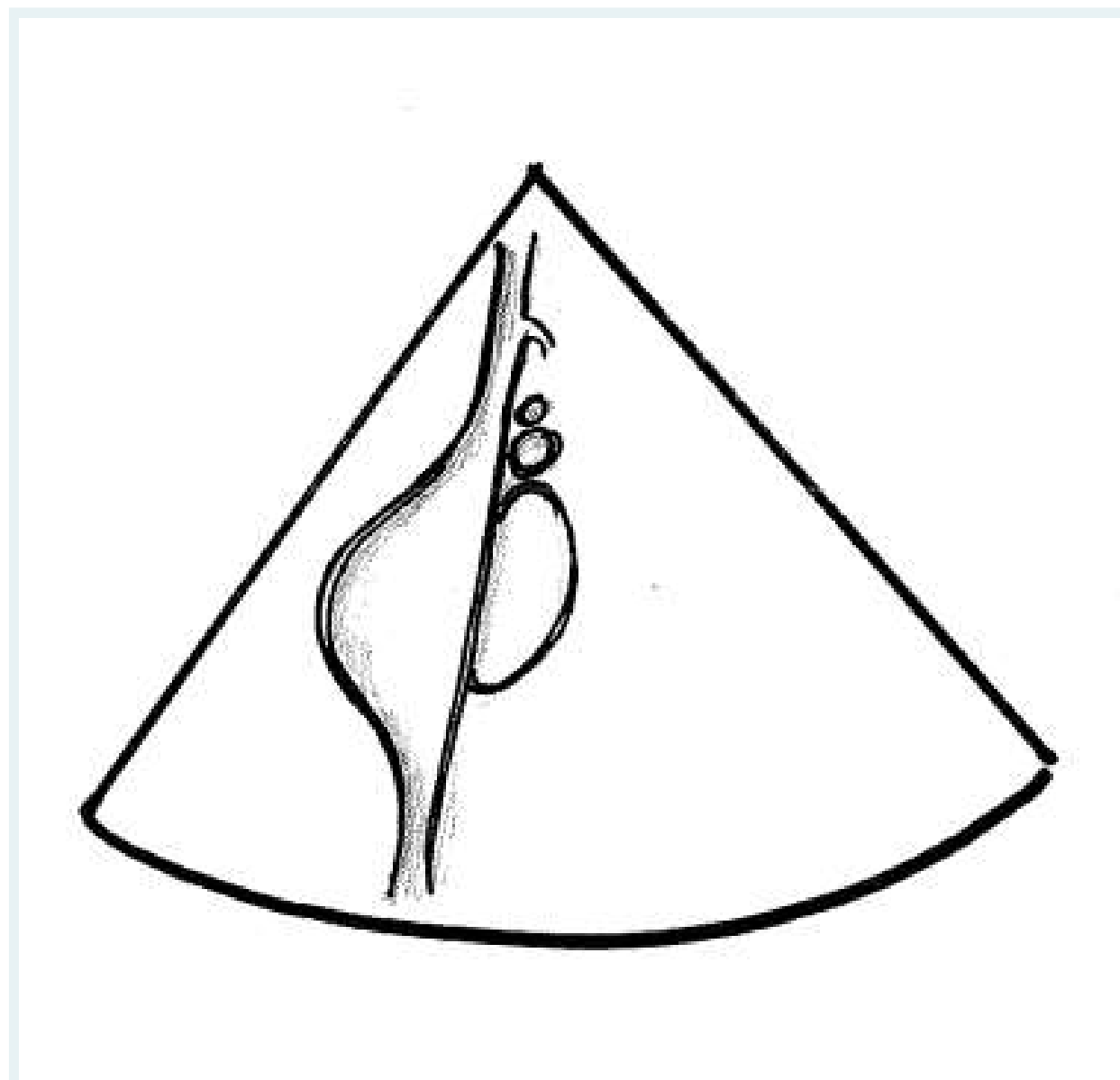

- A. Atrial septum
- B. Left upper pulmonary vein
- C. Aortic arch
- D. Coronary sinus
- E. Hepatic vein

24

This apical 4 chamber view in color presents concern for
(1 Point)

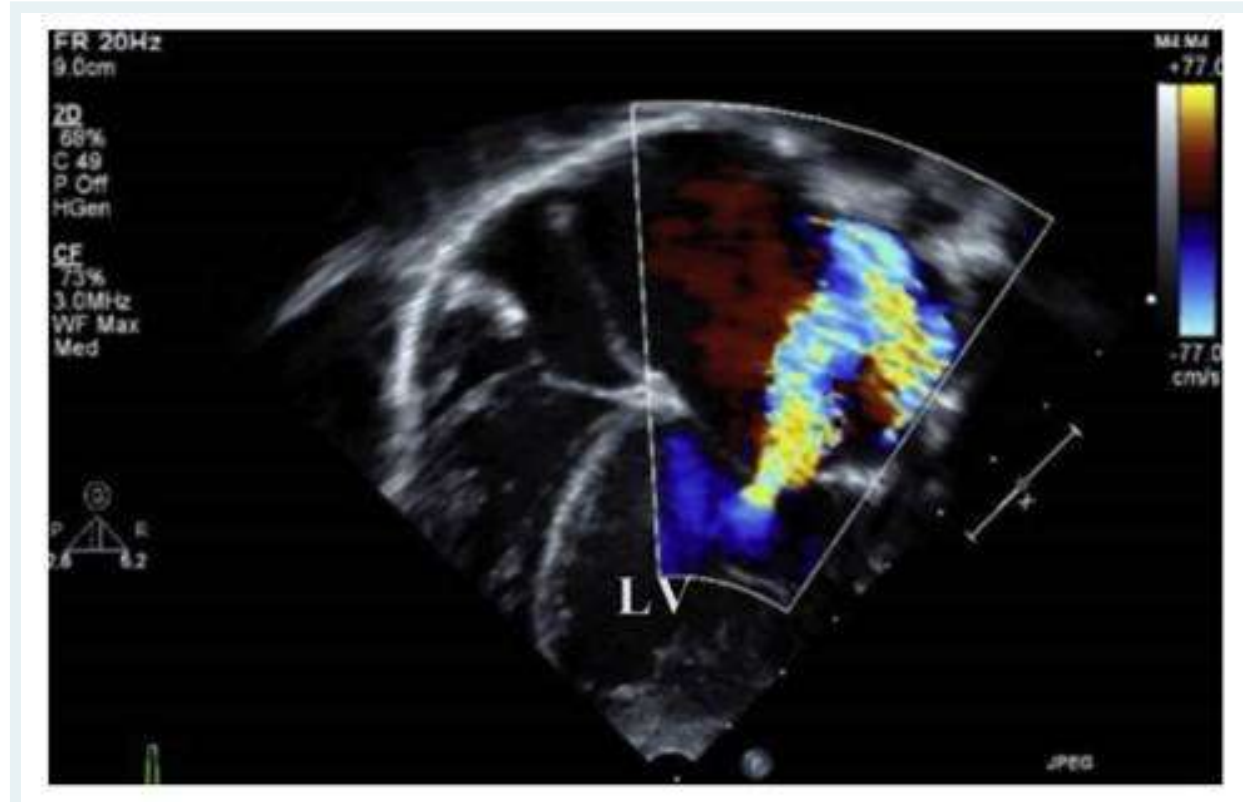

- Mitral valve stenosis
- Mitral valve regurgitation
- Tricuspid valve stenosis
- Tricuspid valve regurgitation

25

Based on this image, pulmonary pressure is estimated to be
(1 Point)

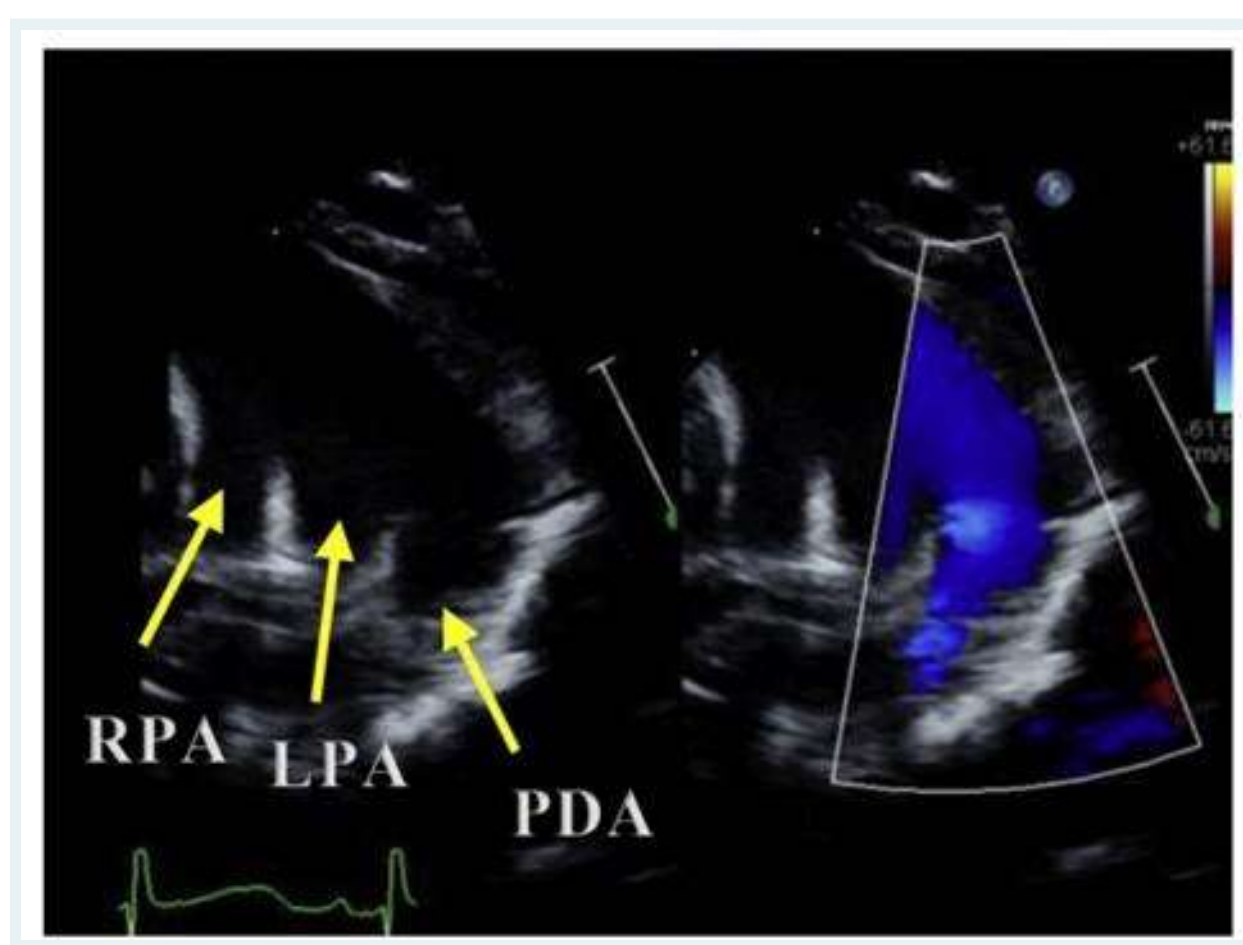

- Subsystemic
- Systemic

◯ Suprasystemic

26

On obtaining this apical 4 chamber view, there is concern for which of the following cardiac pathologies (1 Point)

◯ Coarctation of the aorta

◯ Total anomalous pulmonary venous return

◯ Hypoplastic mitral valve

◯ All of the above

Never give out your password. Report abuse

Microsoft 365

This content is created by the owner of the form. The data you submit will be sent to the form owner. Microsoft is not responsible for the privacy or security practices of its customers, including those of this form owner. Never give out your password.

Microsoft Forms | AI-Powered surveys, quizzes and polls Create my own form

The owner of this form has not provided a privacy statement as to how they will use your response data. Do not provide personal or sensitive information. | Terms of use
