# Supplementary material for: Pediatric Cardiology Echocardiography Curriculum and Workshop for Pediatric Residents in Mwanza, Tanzania-Using Deliberate Practice for Skill Acquisition
Source: Pediatr Cardiol. 2026 Jan 9;47(8):3432–9. doi: 10.1007/s00246-025-04140-9 (PMC13687231; doi:10.1007/s00246-025-04140-9)
Supplement: Supplementary file 3 [file 246_2025_4140_MOESM3_ESM.pdf]

Title: Pediatric Cardiology Echocardiography Curriculum and Workshop for Pediatric Residents in Mwanza, Tanzania – using Deliberate Practice for Skill Acquisition

Authors: Shivani Patel 1, Jennifer Acevedo 1, Angira Patel 1 , Colleen Fant 2, Theophylly Mushi 3, Julieth Kabrigi, 4 Tulla Masoza 4
1- Division of Cardiology, Ann and Robert H. Lurie Children's Hospital of Chicago, 225 East Chicago Ave. Box 21 Chicago, IL. 60611 U.S.A
2 - Division of Emergency Medicine, Ann and Robert H. Lurie Children's Hospital of Chicago, 225 East Chicago Ave. Box 21 Chicago, IL. 60611 U.S.A
3- Department of Pediatric Cardiology, Jakaya Kikwete Cardiac Institute, Tanzania
4 - Department of Pediatrics, Bugando Medical Center, Mwanza, Tanzania
Corresponding Author: Shivani Govind Patel, MBBS, MS, FASE, FACC. Email: shgpatel@luriechildrens.org

Skills Assessment Sheet:

Name: Year of training

Imaging Plane	Attempt 1	Attempt 2	Attempt 3
Subcostal			
Parasternal Long Axis			
Parasternal Short Axis			
Apical			
Suprasternal Notch			

Recognition of normal vs abnormal images:

Question Number	Normal Vs Abnormal
1	
2	
3	
4	
5	
6	
7	
8	
9	
10	
11	
12	
13	
14	
15	
16	
17	
18	
19	
20	
21	
22	
23	
24	
25	
26	
27	
28	
29	
30	
